# Employing urinary biomarkers in predicting renal recovery three months after in-hospital acute kidney injury

**DOI:** 10.1080/0886022X.2025.2522975

**Published:** 2025-07-10

**Authors:** Shih-Ping Hsu, Chiang-Ting Chien

**Affiliations:** ^a^ Department of Internal Medicine, Far Eastern Memorial Hospital, New Taipei, Taiwan; ^b^ School of Life Science, National Taiwan Normal University, Taipei, Taiwan; ^c^ College of Medicine, National Taiwan University, Taipei, Taiwan; ^d^ General Education Center, Lunghwa University of Science and Technology, Taoyuan, Taiwan

**Keywords:** Acute kidney injury, ASSESS-AKI, prognosis, spot urine creatinine-to-osmolality ratio, uromodulin

## Abstract

**Introduction:**

Renal recovery after acute kidney injury (AKI) significantly improves outcomes. This cohort study explored the efficacy of six urinary biomarkers and the spot urine creatinine-to-osmolality ratio (sUCr/Osm) in predicting renal recovery 3 months after in-hospital AKI.

**Methods:**

Data from the Assessment, Serial Evaluation, and Subsequent Sequelae of Acute Kidney Injury study, involving 744 patients with in-hospital AKI, were analyzed. The urinary biomarker with the highest area under the receiver operating characteristic curve (AUC) was selected as the representative for comparison. Renal recovery was defined as an absolute increase of <26.5 μmol/L or a relative elevation of <20% from the baseline serum creatinine (SCr) level at three months postdiagnosis.

**Results:**

Among the 744 patients, 85.6% achieved renal recovery. Uromodulin demonstrated a greater AUC of 0.580 (95% CI: 0.518–0.641) than the other five biomarkers did. With a cutoff of 1,360 ng/mL, uromodulin sensitivity was 0.774 (95% CI: 0.741–0.806). The sUCr/Osm test exhibited high sensitivity (0.881; 95% CI: 0.856–0.906) with a cutoff of 8.84 (sUCr/Osm8.84) and high specificity (0.785; 95% CI: 0.707–0.863) with a cutoff of 21.22 (sUCr/Osm21.22). The positive predictive values of the three methods were approximately 0.880. The performance of these tests in predicting renal recovery based on both criteria in 298 patients with chronic kidney disease was also comparable.

**Conclusion:**

Urinary biomarkers, especially uromodulin, and the sUCr/Osm test may be effective in predicting renal recovery three months after in-hospital AKI. The sUCr/Osm test may offer a more accessible approach for routine use, with sUCr/Osm8.84 demonstrating high sensitivity for screening and sUCr/Osm21.22 exhibiting high specificity for further discrimination.

## Introduction

Acute kidney injury (AKI) is associated with increased risks of mortality and comorbidities in the short term, as well as adverse long-term outcomes, including the development or progression of chronic kidney disease (CKD) and long-term mortality [1,2]. In contrast, recovery of renal function after AKI is associated with better long-term outcomes [3].

The emergence of novel methodologies has advanced the development of predictive tools for recovery from AKI. In addition to machine learning models [4] and risk prediction scores [5], biomarkers that are indicative of kidney injury, inflammation, and tubular health, such as urinary neutrophil gelatinase-associated lipocalin (UNGAL) [6], interleukin-18 (IL-18) [7], and uromodulin (UMOD) [8,9], have been extensively studied. Nevertheless, the use of the spot urine creatinine-to-osmolality ratio (sUCr/Osm), a novel functional marker indicating the relative urinary excretion rate of creatinine to accompanying osmoles independent of actual urine volume or concentrating ability [10], has not yet been widely investigated. Clinically, sUCr/Osm may reflect preserved or recovering renal excretory function soon after acute kidney injury. Higher sUCr/Osm ratios suggest increased renal excretion of creatinine. In addition to its inherent simplicity, convenience, and accessibility, sUCr/Osm has demonstrated its potential for clinical application by indicating the absence of acute kidney disease in outpatients with a single serum creatinine (SCr) measurement [11].

Although the consensus definition of acute kidney injury (AKI) proposed by the Kidney Disease: Improving Global Outcomes (KDIGO) group [12] has gained widespread acceptance and is currently used in clinical practice, there remains a lack of consensus regarding the practical definitions of functional renal recovery after an episode of AKI. In general, the key components to be defined are both the timing of determination and the degree of restoration in terms of the SCr level or estimated glomerular filtration rate (eGFR) [13,14]. The determination of renal recovery has been made at the time of discharge [15,16], seven days postdiagnosis [3,16], or three months (90 days) postdiagnosis [17–20]. However, the criteria for SCr level or eGFR vary considerably. They included a return to within baseline SCr level + 44.2 μmol/L [15], within 50% above baseline SCr level [17], within 25% above baseline SCr level [18], within 120% of a baseline SCr level value [19], eGFR ≥ 90% of baseline eGFR [20], a decrease in SCr level ≥ 44.2 μmol/L or 25% from the maximum value [3], or the absence of any KDIGO stage of AKI according to the SCr criteria [16]. Nonetheless, one essential factor that can lead to considerable discrepancies when applying the criteria of absolute values or relative proportions of SCr changes to defined renal recovery, namely, the baseline SCr level or the preexisting CKD status, is often overlooked. Theoretically, the application of criteria on the basis of absolute SCr changes will result in a greater rate of renal recovery in patients with lower baseline SCr levels or without preexisting CKD than the use of criteria on the basis of the relative proportion of changes.

Therefore, the aim of this exploratory study was to compare the efficacy of known urinary biomarker tests with that of sUCr/Osm in predicting renal recovery three months after the diagnosis of in-hospital AKI. The analysis was conducted in consideration of the criteria based on absolute or relative changes in SCr values, as well as the presence of preexisting CKD, for the purpose of sensitivity analysis.

## Materials and methods

### Study population

The dataset used in the current study was derived from the Assessment, Serial Evaluation, and Subsequent Sequelae of Acute Kidney Injury (ASSESS-AKI) Study [3], and the original datasets were provided by the National Institute of Diabetes and Digestive and Kidney Diseases (NIDDK) Central Repository upon request.

The ASSESS-AKI study was designed to examine the influence of an in-hospital AKI episode on subsequent mortality and renal outcomes. Written informed consent was obtained from all participants at their respective institutions, and the study was approved by the respective institutional review boards [21,22]. The criteria for enrollment and the specifics of the project have been published [21,22]. In brief, the study recruited individuals aged 18– 89 years who had been hospitalized between December 2009 and February 2015. The participants were scheduled for an outpatient follow-up visit at 3 months postdischarge. The diagnosis and severity assessment of AKI were based on the peak SCr levels observed during hospitalization compared with the patient’s baseline level. The baseline SCr level was determined as the closest value to the index hospitalization on an outpatient, nonemergency occasion within 7–365 days before admission or 7 days before nonurgent cardiac surgery. Owing to limitations in the quality of data collection, the urine output criteria for patients with AKI were not applied. More than one-third of the participants met the criteria for AKI lasting 48 h or more. Additionally, over one-third of the participants demonstrated a relative increase in SCr of 100% or more, indicative of stage 2 AKI or higher.

**Figure 1.**
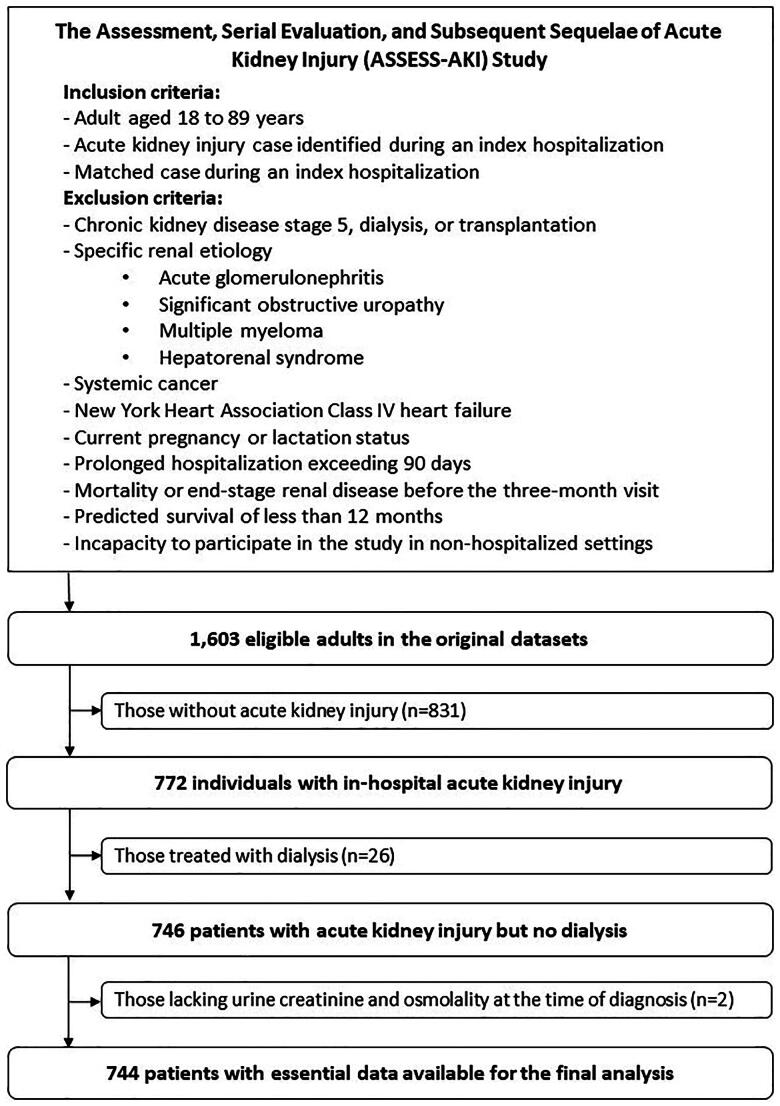
The sampling flow chart.

The original datasets included data from 1,603 adults. After the exclusion of 831 matched control patients without AKI, 26 patients receiving in-hospital dialysis, and two patients lacking essential data, the final dataset for analysis consisted of 744 patients with in-hospital AKI. Figure 1 depicts the sampling process. The current study protocol was reviewed and granted an exemption from requiring ethics approval by the Research Ethics Review Committee, Far Eastern Memorial Hospital (approval numbers 111176-W and 113248-W). Patient consent was waived, as this study was based on publicly available, deidentified data. This study was conducted in accordance with the Declaration of Helsinki.

### Demographics, comorbidities, and medication use

The date of birth, sex, and race were provided by the participants during in-hospital enrollment. Comorbid conditions were obtained through medical records or self-reports. Preexisting CKD was defined as a baseline eGFR of <60 mL/min/1.73 m^2^, according to the 2009 CKD-EPI equation. The smoking status and the insults associated with AKI were also documented.

At the 3-month outpatient visit following discharge, participants’ height and body weight (BW) were measured while standing or were self-reported. All medications taken within the previous 30 days were recorded.

### Blood and urine sampling

Nonfasting samples were collected within four days following the diagnosis of AKI and subsequently analyzed by the Central Lab at the University of Minnesota. SCr levels were measured using an isotope-dilution mass spectrometry (IDMS)-traceable assay.

### Urinary biomarker measurement

The urinary biomarkers measured included NGAL, kidney injury molecule-1 (KIM-1), IL-18, monocyte chemoattractant protein 1 (MCP-1), UMOD, and chitinase 3-like 1 (YKL-40) [23]. The urine samples were subjected to centrifugation at 1000 × g for 10 min at room temperature within 30 min of collection. Otherwise, the samples were refrigerated until processing at six hours postcollection. Following processing, the samples were aliquoted, frozen, and stored at −70 °C until measurement. The assays used for the measurement of these biomarkers have been previously published [24].

### Renal functional recovery

In the current study, the definition of functional renal recovery was the resolution and absence of any KDIGO stage of AKI according to the SCr criteria. In other words, the final absolute elevation in SCr from baseline was <26.5 μmol/L. An alternative definition based on a relative increase in SCr from baseline <20% was also adopted for comparison. The determination was made at the first outpatient visit, which was scheduled for three months postdischarge.

### Representative urinary biomarkers and cutoffs

To perform a more concise comparison with the novel sUCr/Osm, only the best biomarker was selected as the representative one through evaluation of the efficacy of the six urinary biomarkers in predicting renal recovery. This determination was based on the area under the curve (AUC) values in the respective receiver operating characteristic (ROC) curves of the six biomarkers tested, with all individuals having in-hospital AKI. The cutoff values of the representative biomarker was subsequently determined by identifying the point at which the Youden index reached its maximum value. The cutoff of the sUCr/Osm test was set using the same method.

### Statistical analysis

The ASSESS-AKI study datasets are provided in SAS files. The SUBJ_ID of each participant was used as the identifier to merge the relevant datasets. The current analysis was conducted using IBM SPSS Statistics 22.0 (New York, USA). MedCalc 23.1.7 (Ostend, Belgium) was used to plot the precision–recall (PR) curves [25] and calculate the AUC.

Unless otherwise indicated, the data are reported as the mean ± standard deviation (SD), median [interquartile range], or a number (%), as appropriate. If applicable, differences were analyzed using Student’s t test, the chi-square test, or one-way analysis of variance (ANOVA) with the Bonferroni method as the *post hoc* test. The statistical significance of the urinary biomarkers for the prediction of renal recovery was evaluated using the chi-square test. A *P* value of <0.05 was considered statistically significant for two-tailed tests, unless otherwise noted.

## Results

### Patient characteristics

The analyzed dataset included information from all 744 patients who experienced in-hospital AKI, with a mean age of 64 ± 13 years. Among them, 32.9% were female, and 15.2% were black. The mean body weight (BW) was 93 ± 25 kg. The prevalence of hypertension was 78.8%, and that of diabetes mellitus (DM) was 50.1%. The proportion of individuals receiving angiotensin-converting enzyme inhibitors (ACEIs) or angiotensin II receptor blockers (ARBs) was 52.3%, whereas that of those receiving loop diuretics was 37.2%. The prevalence of the use of nonsteroidal anti-inflammatory drugs (NSAIDs) was 5%. Sodium–glucose transport protein 2 inhibitors (SGLT2is) were not yet available at the time of the original study. More details are shown in Table 1.

**Table 1.** Characteristics of all patients with in-hospital acute kidney injury, stratified and compared according to renal recovery or not.

	All patients	Recovery	Nonrecovery	
	*N* = 744	*N* = 637	*N* = 107	*P* *
Age, year	63.9 ± 12.8	63.8 ± 12.6	64.5 ± 13.7	0.648
Female sex	245 (32.9)	202 (31.7)	43 (40.2)	0.084
Body weight, kg	92.6 ± 25.3	93.1 ± 25.3	90.0 ± 25.2	0.253
BMI, kg/m^2^	31.6 ± 8.4	31.6 ± 8.5	31.4 ± 8.0	0.739
Race				0.678
Black	113 (15.2)	92 (14.4)	21 (19.6)	
White	588 (79.0)	512 (80.4)	76 (71.0)	
Other Races	43 (5.7)	33 (5.2)	10 (9.3)	
Non-smoker	298 (40.1)	250 (43.5)	48 (49.3)	0.427
Comorbidity				
CKD	298 (40.1)	243 (38.1)	55 (51.4)	**0.009**
Hypertension	586 (78.8)	500 (78.5)	86 (80.4)	0.660
Diabetes mellitus	373 (50.1)	306 (48.0)	67 (62.6)	**0.005**
CAD	358 (48.1)	302 (47.4)	56 (52.3)	0.345
CHF	199 (26.7)	158 (24.8)	41 (38.3)	**0.003**
Chronic lung diseases	180 (24.2)	158 (24.8)	22 (20.6)	0.343
Chronic liver diseases	36 (4.8)	31 (4.9)	5 (4.7)	0.931
In-hospital AKI				0.405
Stage 1	553 (74.3)	479 (75.2)	74 (69.2)	
Stage 2	119 (16.0)	99 (15.5)	20 (18.7)	
Stage 3	72 (9.7)	59 (9.3)	13 (12.1)	
Related insult to AKI				
Medication	613 (82.4)	524 (82.3)	89 (83.2)	0.818
Contrast use	47 (6.3)	41 (6.4)	6 (5.6)	0.744
Cardiac event	135 (18.1)	113 (17.7)	22 (20.6)	0.483
Sepsis	106 (14.2)	90 (14.1)	16 (15.0)	0.821
Major surgery	302 (40.6)	265 (41.6)	37 (34.6)	0.171
Unknown	80 (10.8)	68 (10.7)	12 (11.2)	0.868
ICU stay	525 (70.6)	454 (71.3)	71 (66.4)	0.302
Medication use				
ACEI	268 (36.0)	233 (36.6)	35 (32.7)	0.441
ARB	121 (16.3)	97 (15.2)	24 (22.4)	0.062
NSAID	37 (5.0)	32 (5.0)	5 (4.7)	0.877
Loop diuretics	277 (37.2)	213 (33.4)	64 (59.8)	**<0.001**
Serum Creatinine, μmol/L				
Baseline	109 ± 48	106 ± 46	125 ± 58	**0.002**
Date of AKI	202 ± 120	196 ± 116	241 ± 139	**0.002**
3-mo post-diagnosis	114 ± 62	103 ± 42	185 ± 100	**0.002**
eGFR, mL/min/1.73 m^2^				
Baseline	67.2 ± 25.8	68.6 ± 25.6	58.8 ± 25.3	**<0.001**
Date of AKI	35.5 ± 16.5	36.6 ± 16.6	29.1 ± 14.3	**<0.001**
3-mo post-diagnosis	65.7 ± 26.7	70.6 ± 25.2	37.0 ± 15.5	**<0.001**
Urine biochemistry				
Creatinine, μmol/L	8425 ± 5357	8575 ± 5384	7523 ± 5083	0.59
Osm, mOsm/Kg	470 ± 165	478 ± 168	425 ± 136	**0.001**
UCr/Osm	17.68 ± 8.84	17.68 ± 8.84	16.80 ± 8.84	0.403
UACR, mg/mmol	5.3 (2.0–13.5)	5.1 (2.0–12.0)	8.6 (2.9–71.0)	**<0.001**
Urinary biomarkers				
NGAL, ng/mL	65 (28–179)	61 (28–163)	119 (36–268)	0.126
KIM-1, pg/mL	2727 (1174–5857)	2717 (1166–6045)	2912 (1232–5623)	0.712
IL-18, pg/mL	40 (18–89)	40 (18–90)	21 (12–48)	0.613
MCP-1, pg/mL	480 (234–1082)	482 (234–1079)	469 (244–1108)	0.289
UMOD, ng/mL	2256 (1376–4218)	2287 (1435–4262)	1930 (950–3929)	**0.045**
YKL-40, pg/mL	1220 (383–4619)	1193 (393–4147)	1348 (318–12408)	**0.043**

Note: Data are presented as mean ± standard deviation, number (%), or median [interquartile range].

* Comparisons between the groups of ‘Recovery’ and ‘Nonrecovery’ using the Student’s *t*-test, chi-square test, or Fisher’s exact test as appropriate.

ACEI: angiotensin converting enzyme inhibitor; AKI: acute kidney injury; ARB: angiotensin II receptor blocker; BMI: body mass index; CAD: coronary artery disease; CHF: chronic heart failure; CKD: chronic kidney disease; Dis: disease; eGFR: estimated glomerular filtration rate; ICU: intensive care unit; IL-18: interleukin-18; KIM-1: Kidney Injury Molecule-1; MCP-1: monocyte chemoattractant protein 1; NGAL: neutrophil gelatinase-associated lipocalin; NSAID: non-steroidal anti-inflammatory drug; Osm: osmolality; UCr/Osm: urine creatinine-to-osmolality ratio; UACR: urine albumin to creatinine ratio; UMOD: uromodulin; YKL-40: chitinase 3-like 1.

To convert creatinine to mg/dL, divided by 88.4; UACR to mg/g, 0.113.

Among the 744 enrolled patients, 637 (85.6%) were determined to have achieved renal recovery according to the definition of the absolute change criterion of SCr level at the outpatient visit at three months postdischarge. The median interval between the diagnosis of AKI and the determination was 92 (79–110) days.

Compared with the group with renal recovery, the group without renal recovery had a greater prevalence of preexisting CKD, DM, congestive heart failure (CHF), and current use of loop diuretics (Table 1).

Among the 744 patients, 298 presented with preexisting CKD. The characteristics of these patients are presented in Table 2. Among the 298 patients with preexisting CKD, 243 (81.5%) achieved renal recovery according to the absolute change criterion. Compared with the other 243 patients who achieved renal recovery, the 55 patients who did not achieve renal recovery presented a greater prevalence of DM and a higher rate of loop diuretic use (Table 2).

**Table 2.** Characteristics of the patients with acute kidney injury on preexisting chronic kidney disease, stratified and compared according to renal recovery or not.

	Patients with CKD	Recovery	Nonrecovery	
	*N* = 298	*N* = 243	*N* = 55	P*
Age, year	68.4 ± 11.3	69.0 ± 10.4	65.6 ± 14.4	0.105
Female sex	120 (40.3)	96 (39.5)	24 (43.6)	0.573
Body weight, kg	91.9 ± 24.9	92.4 ± 24.7	89.5 ± 26.0	0.431
BMI, kg/m^2^	31.9 ± 8.2	32.0 ± 8.0	31.5 ± 8.8	0.719
Race				0.623
Black	49 (16.4)	38 (15.6)	11 (20.0)	
White	226 (75.8)	190 (78.2)	36 (65.5)	
Other Races	23 (7.7)	15 (6.2)	8 (14.5)	
Non-smoker	129 (43.3)	106 (43.6)	23 (41.8)	0.807
Comorbidity				
Hypertension	254 (85.2)	207 (85.2)	47 (85.5)	0.959
Diabetes mellitus	179 (60.1)	139 (57.2)	40 (72.7)	**0.034**
CAD	166 (55.7)	136 (56.0)	30 (54.5)	0.848
CHF	115 (38.6)	88 (36.2)	27 (49.1)	0.076
Chronic lung diseases	81 (27.2)	65 (26.7)	16 (29.1)	0.724
Chronic liver diseases	16 (5.4)	15 (6.2)	1 (1.84)	0.321
In-hospital AKI				0.159
Stage 1	252 (84.6)	207 (85.2)	45 (81.8)	
Stage 2	29 (9.7)	25 (10.3)	4 (7.3)	
Stage 3	17 (5.7)	11 (4.3)	6 (10.9)	
Related insult to AKI				
Medication	251 (84.2)	202 (83.1)	49 (89.1)	0.273
Contrast use	14 (4.7)	11 (4.5)	3 (5.5)	0.728
Cardiac event	53 (17.8)	41 (16.9)	12 (21.8)	0.386
Sepsis	27 (9.1)	23 (9.5)	4 (7.3)	0.796
Major surgery	103 (34.6)	88 (36.2)	15 (27.3)	0.208
Unknown	34 (11.4)	30 (12.3)	4 (7.3)	0.285
ICU stay	201 (67.4)	167 (68.7)	34 (61.8)	0.324
Medication use				
ACEI	92 (30.9)	78 (32.1)	14 (25.5)	0.335
ARB	57 (19.1)	44 (18.1)	13 (23.6)	0.346
NSAID	10 (3.4)	9 (3.7)	1 (1.8)	0.695
Loop diuretics	165 (55.4)	127 (52.3)	38 (69.1)	**0.023**
Serum Creatinine, μmol/L				
Baseline	148 ± 53	144 ± 51	164 ± 57	**0.022**
Date of AKI	247 ± 123	237 ± 118	288 ± 136	**0.005**
3-mo post-diagnosis	149 ± 69	133 ± 51	225 ± 86	**<0.001**
eGFR, mL/min/1.73 m^2^				
Baseline	42.3 ± 12.0	43.2 ± 12.1	38.2 ± 11.1	**0.005**
Date of AKI	24.8 ± 9.3	25.6 ± 9.3	21.2 ± 8.4	**0.001**
3-mo post-diagnosis	44.7 ± 17.3	48.8 ± 16.2	26.3 ± 7.7	**<0.001**
Urine biochemistry				
Creatinine, μmol/L	7903 ± 4606	8274 ± 4685	6285 ± 3881	**0.004**
Osm, mOsm/Kg	434 ± 128	443 ± 131	392 ± 107	**0.003**
UCr/Osm	17.68 ± 8.84	18.56 ± 8.84	15.91 ± 8.84	**0.043**
UACR, mg/mmol	7.0 (2.9–27.7)	5.8 (2.4–21.4)	16.1 (6.2–200.0)	**0.002**
Urinary biomarkers				
NGAL, ng/mL	81 (29–238)	73 (29–197)	148 (29–318)	0.393
KIM-1, pg/mL	1979 (986–4574)	1977 (949–4568)	1992 (1092–4855)	0.762
IL-18, pg/mL	33 (15–73)	33 (15–74)	33 (12–71)	0.215
MCP-1, pg/mL	379 (195–769)	380 (165–772)	377 (213–716)	0.651
UMOD, ng/mL	1942 (1198–3205)	2034 (1276–3494)	1493 (764–2341)	**0.036**
YKL-40, pg/mL	1174 (306–6017)	1126 (302–4884)	1216 (338–14,074)	0.165

Note: Data are presented as mean ± standard deviation, number (%), or median [interquartile range].

*Comparisons between the groups of ‘Recovery’ and ‘Nonrecovery’ using the Student’s *t*-test, chi-square test, or Fisher’s exact test as appropriate.

ACEI: angiotensin converting enzyme inhibitor; AKI: acute kidney injury; ARB: angiotensin II receptor blocker; BMI: body mass index; CAD: coronary artery disease; CHF: chronic heart failure; CKD: chronic kidney disease; Dis: disease; eGFR: estimated glomerular filtration rate; ICU: intensive care unit; IL-18: interleukin-18; KIM-1: Kidney Injury Molecule-1; MCP-1: monocyte chemoattractant protein 1; NGAL: neutrophil gelatinase-associated lipocalin; NSAID: non-steroidal anti-inflammatory drug; Osm: osmolality; UCr/Osm: urine creatinine-to-osmolality ratio; UACR: urine albumin to creatinine ratio; UMOD: uromodulin; YKL-40: chitinase 3-like 1.

To convert creatinine to mg/dL, divided by 88.4; UACR to mg/g, 0.113.

### Blood and urine tests

Among the 744 patients who experienced in-hospital AKI, the baseline SCr level was 109 ± 48 μmol/L. At the time of AKI diagnosis and 3 months postdiagnosis, the SCr levels were 202 ± 120 and 114 ± 62 μmol/L, respectively. The levels of UMOD and sUCr/Osm within four days after the diagnosis of AKI were 2,256 (1,376–4,218) ng/mL and 17.68 ± 8.84, respectively. Further comparison of the 637 patients who exhibited renal recovery as defined by the absolute change criterion and the 107 patients who did not demonstrate renal recovery revealed that the former group exhibited lower SCr levels at all three time points: baseline, AKI diagnosis, and three months postdiagnosis. Additionally, they demonstrated higher levels of urine osmolality and UMOD, as well as lower levels of urine albumin-to-creatinine and YKL-40. More details are shown in Table 1.

When the 243 patients with preexisting CKD and renal recovery were compared with the 55 patients with preexisting CKD but no renal recovery, the former group presented lower SCr levels and higher eGFRs from baseline to three months postdiagnosis. In addition, the former group presented higher urine levels of creatinine, osmolality, UCr/Osm, and UMOD, whereas the UACR was lower (Table 2).

### Test performance in predicting renal recovery

**Figure 2.**
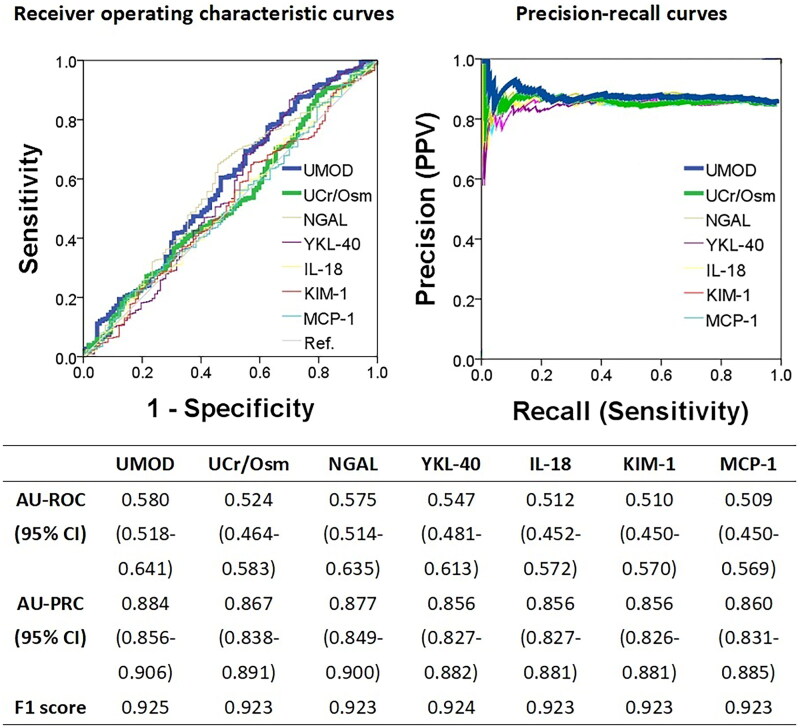
Areas under receiver operating characteristic curves and precision-recall curves for urinary biomarkers in predicting renal recovery three months after in-hospital acute kidney injury, defined by a serum creatinine increase of less than 26.5 μmol/L from baseline. Abbreviations: AU-PRC: area under precision-recall curve; AU-ROC: area under receiver operating characteristic curve; CI: confidence interval; F1 score: the harmonic mean (a kind of average) of precision and recall; IL-18: interleukin-18; KIM-1: kidney injury molecule-1; MCP-1: monocyte chemoattractant protein 1; UNGAL: urine neutrophil gelatinase-associated lipocalin; UCr/osm: urine creatinine-to-osmolality ratio; UMOD: uromodulin; YKL-40: chitinase 3-like 1.

As described in the Methods section, the AUC values of six known urinary biomarkers for predicting renal recovery based on the definitions of the absolute change criterion were initially evaluated in all 744 participants with in-hospital AKI. UMOD exhibited the highest AUC of 0.580 (95% CI: 0.518–0.641), whereas the AUC for sUCr/Osm was 0.524 (95% CI: 0.464–0.583). Further details are displayed in Figure 2. Considering that the ROC curve method may have been greatly compromised by the substantial discrepancy in numbers between patients who achieved renal recovery and patients who did not achieve renal recovery (637 vs. 107), the PR curve method was additionally adopted to assess the test performance. As shown in Figure 2, UMOD still exhibited the greatest AUC value of the PR curve at 0.884 (95% CI: 0.856–0.906), whereas the value for sUCr/Osm was 0.867 (95% CI: 0.838–0.891). Accordingly, UMOD was identified as a representative biomarker for subsequent analysis, with a cutoff value of 1,360 ng/mL determined using the Youden index. A test result of UMOD ≥ 1,360 ng/mL was considered positive, whereas a result below this threshold was regarded as negative. After extensive consideration, two cutoff values for the sUCr/Osm test were identified as candidates, namely, 8.84 and 21.22, on the basis of their corresponding Youden index values, which were closely aligned. The rationale underlying this decision will be discussed in detail in the Discussion. Therefore, in the sUCr/Osm test with 8.84 as the cutoff (sUCr/Osm8.84), a value of sUCr/Osm ≥ 8.84 was designated as positive, whereas a value below this threshold was classified as negative. Similarly, when the sUCr/Osm test with 21.22 as the cutoff (sUCr/Osm21.22) was employed, a value of sUCr/Osm ≥ 21.22 was designated as positive, whereas a value below this threshold was classified as negative.

The performance metrics of the above three tests for predicting renal recovery based on the definition of the absolute change criterion are presented in Table 3. In all 744 patients with in-hospital AKI, the sUCr/Osm8.84 test exhibited the highest sensitivity (0.881; 95% CI: 0.856–0.906), whereas the sUCr/Osm21.22 test demonstrated the highest specificity (0.785; 95% CI: 0.707–0.863). As the renal recovery rate was 85.6%, the positive predictive value (PPV) of the three tests was found to be approximately 0.880. Upon further examination of the three tests in the 298 patients with preexisting CKD, the sUCr/Osm8.84 test once again demonstrated the highest sensitivity (0.901; 95% CI: 0.864–0.939), whereas the sUCr/Osm21.22 test exhibited the highest specificity (0.873; 95% CI: 0.785–0.961). With a renal recovery rate of 81.5%, the PPVs ranged from 0.842 to 0.914.

**Table 3.** Test performance metrics of the urinary tests in predicting renal recovery three months after in-hospital acute kidney injury, defined by a serum creatinine increase of less than 26.5 μmol/L from baseline.

		All patients (*n* = 744)			Patients with CKD (*n* = 298)		
		Recovery	Nonrecovery		Recovery	Nonrecovery	
Test		*n* = 637	*n* = 107	*P*	*n* = 243	*n* = 55	*P*
UMOD	**+**	493	67	0.001	176	28	0.002
	**-**	144	40		67	27	
sUCr/Osm 8.84	**+**	561	85	0.015	219	41	0.002
	**−**	76	22		24	14	
sUCr/Osm 21.22	**+**	168	23	0.285	74	7	0.008
	**−**	469	84		169	48	
		UMOD	sUCr/Osm 8.84	sUCr/Osm 21.22	UMOD	sUCr/Osm 8.84	sUCr/Osm 21.22
Sensitivity (95% CI)		0.774 (0.741–0.806)	0.881 (0.856–0.906)	0.264 (0.230–0.298)	0.724 (0.668–0.780)	0.901 (0.864–0.939)	0.305 (0.247–0.362)
Specificity (95% CI)		0.374 (0.282–0.466)	0.206 (0.129–0.282)	0.785 (0.707–0.863)	0.491 (0.359–0.623)	0.255 (0.139–0.370	0.873 (0.785–0.961)
PPV (95% CI)		0.880 (0.853–0.907)	0.868 (0.842–0.894)	0.880 (0.833–0.926)	0.863 (0.816–0.910)	0.842 (0.798–0.887)	0.914 (0.852–0.975)
NPV (95% CI)		0.217 (0.158–0.277)	0.224 (0.142–0.307)	0.152 (0.122–0.182)	0.287 (0.196–0.379)	0.368 (0.215–522)	0.221 (0.166–0.276)

Note. Cutoffs for the tests: For UMOD: ≥1,360 ng/mL, (+); otherwise, (−). For sUCr/Osm8.84: ≥8.84 (+); otherwise, (−). For sUCr/Osm21.22: ≥21.22 (+); otherwise, (−).

CKD: chronic kidney disease; CI: confidence interval; NPV: negative predictive value; PPV: positive predictive value; sUCr/Osm: spot urine creatinine-to-osmolality ratio; UMOD: uromodulin.

**Figure 3.**
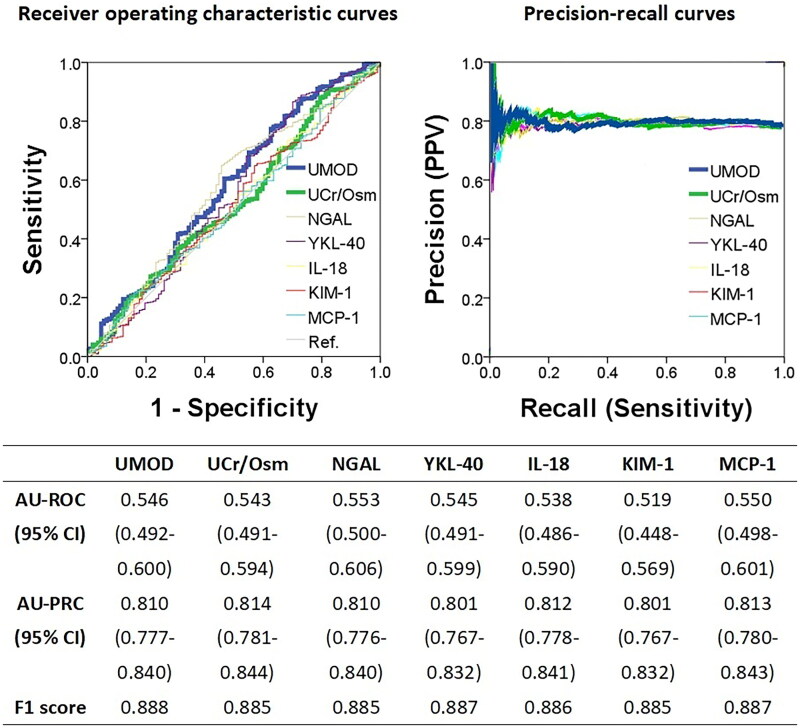
Areas under receiver operating characteristic curves and precision-recall curves for urinary biomarkers in predicting renal recovery three months after in-hospital acute kidney injury, defined by a serum creatinine increase of less than 20% from baseline. AU-PRC: area under precision-recall curve; AU-ROC: area under receiver operating characteristic curve; CI: confidence interval; F1 score: the harmonic mean (a kind of average) of precision and recall; IL-18: interleukin-18; KIM-1: kidney injury molecule-1; MCP-1: monocyte chemoattractant protein 1; UNGAL: urine neutrophil gelatinase-associated lipocalin; UCr/osm: urine creatinine-to-osmolality ratio; UMOD: uromodulin; YKL-40: chitinase 3-like 1.

Figure 3 shows the AUC values of the ROC and PR curves for the six urinary biomarkers and sUCr/Osm in predicting renal recovery, which are based on the definition of the relative change criterion. NGAL had the highest AUC value of the ROC curves (0.553, 95% CI: 0.500–0.606), whereas the values for UMOD and sUCr/Osm were 0.546 (95% CI: 0.492–0.600) and 0.543 (95% CI: 0.491–0.594), respectively. The AUC value of the PR curve for SUCr/Osm was the largest at 0.814 (95% CI: 0.781–0.844). Considering that there were no significant differences among the AUC values of the ROC and PR curves for the six urinary biomarkers and for the sake of consistency and conciseness in further comparisons, UMOD was still adopted as a representative. Table 4 presents the performance metrics of the three tests, UMOD, sUCr/Osm8.84, and sUCr/Osm21.22, used to predict renal recovery on the basis of the definition of the relative change criterion. Similarly, in all 744 patients with in-hospital AKI, the sUCr/Osm8.84 test yielded the highest sensitivity (0.886; 95% CI: 0.861–0.912), whereas the sUCr/Osm21.22 test achieved the highest specificity (0.799; 95% CI: 0.735–0.862). As the renal recovery rate was 79.3%, the PPVs of the three tests ranged from 0.810 to 0.838. In the 298 patients with preexisting CKD, the sUCr/Osm8.84 test yielded the highest sensitivity (0.899; 95% CI: 0.861–0.936), whereas the sUCr/Osm21.22 test achieved the highest specificity (0.922; 95% CI: 0.848–0.995). With a renal recovery rate of 82.9%, the PPVs ranged from 0.854 to 0.951.

**Table 4.** Test performance metrics of the urinary tests in predicting renal recovery three months after in-hospital acute kidney injury, defined by a serum creatinine increase of less than 20% from baseline.

		All patients (*n* = 744)			Patients with CKD (*n* = 298)		
		Recovery	Nonrecovery		Recovery	Nonrecovery	
Test		*n* = 590	*n* = 154	*P*	*n* = 247	*n* = 51	*P*
UMOD	**+**	455	105	0.022	179	25	0.001
	**−**	135	49		68	26	
sUCr/Osm	**+**	523	123	0.004	222	38	0.003
8.84	**−**	67	31		25	13	
sUCr/Osm	**+**	160	31	0.077	77	4	0.001
21.22	**−**	430	123		170	47	
		UMOD	sUCr/Osm 8.84	sUCr/Osm 21.22	UMOD	sUCr/Osm 8.84	sUCr/Osm 21.22
Sensitivity (95% CI)		0.771 (0.737–0.805)	0.886 (0.861–0.912)	0.271 (0.235–0.307)	0.725 (0.669–0.780)	0.899 (0.861–0.936)	0.312 (0.254–0.370)
Specificity (95% CI)		0.318 (0.245–0.392)	0.201 (0.138–0.265)	0.799 (0.735–0.862)	0.510 (0.373–0.647)	0.255 (0.135–0.375)	0.922 (0.848–0.995)
PPV (95% CI)		0.813 (0.780–0.845)	0.810 (0.779–0.840)	0.838 (0.785–0.890)	0.877 (0.832–0.922)	0.854 (0.811–0.897)	0.951 (0.903–0.998)
NPV (95% CI)		0.266 (0.202–0.330)	0.316 (0.224–0.408)	0.222 (0.188–0.257)	0.277 (0.186–0.367)	0.342 (0.191–0.493)	0.217 (0.162–0.271)

**Note:** Cutoffs for the tests: For UMOD: ≥1,360 ng/mL, (+); otherwise, (−). For sUCr/Osm8.84: ≥ 8.84 (+); otherwise, (−). For sUCr/Osm21.22: ≥ 21.22 (+); otherwise, (−).

CKD: chronic kidney disease; CI: confidence interval; NPV: negative predictive value; PPV: positive predictive value; sUCr/Osm: spot urine creatinine-to-osmolality ratio; UMOD: uromodulin.

## Discussion

The results suggest that the urinary UMOD test may serve as a reliable predictor of renal recovery after in-hospital AKI, with high PPVs (0.810 to 0.951), under various conditions, including absolute and relative SCr changes and preexisting CKD. The sUCr/Osm21.22 test presented the highest PPV across all conditions. Additionally, the sUCr/Osm8.84 test demonstrated the highest sensitivity, whereas the sUCr/Osm21.22 test exhibited the highest specificity.

The predictive capacity of the six urinary biomarkers examined in the original ASSESS-AKI study for renal recovery (using disparate definitions from those employed in the current study) has been evaluated for clinical application and reviewed elsewhere [6–9,26,27]. In the present study, the AUC values for the ROC curves of six urinary biomarkers and sUCr/Osm in predicting renal recovery ranged from 0.509 to 0.580. In contrast, the PR curves had AUC values between 0.801 and 0.884, with F1 scores ranging from 0.885 to 0.925. (Figures 2 and 3). The PR curves excel in highly imbalanced datasets by highlighting the positive class, making them ideal for applications such as information retrieval and medical diagnosis. A higher AUC value in the PR curves indicates better performance, whereas a higher F1 score signifies a better balance between precision and recall [25]. Notably, C–C motif chemokine ligand 14 [28], not included in the ACCESS-AKI study and therefore not included in the current analysis, was also proposed as a potential urinary biomarker for predicting clinical renal recovery [29] and was supported by a meta-analysis [30]. With respect to UMOD, each standard deviation increase in UMOD from baseline at in-hospital AKI diagnosis to 12 months postdischarge was associated with a 40% reduction in the risk of developing CKD thereafter [9]. In the present study, UMOD presented the highest AUC value among the six candidate biomarkers for predicting renal recovery on the basis of the absolute change criterion. With a cutoff of 1,360 ng/mL, the UMOD test exhibited high PPVs, with sensitivity values ranging from 0.724 to 0.774 across the conditions. Consequently, the urinary UMOD test is proposed as a reliable tool for predicting renal recovery at three months postdiagnosis. However, the comparative performance of the sUCr/Osm8.84 and sUCr/Osm21.22 tests was also demonstrated, with noninferior PPVs under identical conditions. The greater sensitivity and specificity of the sUCr/Osm test, along with its easy accessibility, may render it a more practical tool for predicting renal recovery following in-hospital AKI.

The definition and criteria for renal recovery following AKI remain debatable and depend on the intended clinical application. To predict in-hospital outcomes and ensure that patients receive necessary care during the acute phase, it may be preferable to utilize the reverse criteria for AKI, which can be determined quickly. In contrast, to avoid excessive concern and facilitate a smooth transition to chronic care, the degree of recovery to baseline renal function after an adequate observation period, such as three months (concordant with the K/DOQI CKD criteria [31]), is an appropriate benchmark. Given the current study’s focus on the latter application, the definition employed was an absolute change from the baseline SCr level <26.5 μmol/L or a relative increase < 20%, determined at three months postdischarge, approximately equivalent to three months postdiagnosis of in-hospital AKI.

For an AKI patient with a baseline SCr level of 132.6 μmol/L, the application of either absolute or relative change in SCr as the criterion for determining renal recovery yielded no difference. However, when the baseline SCr level is < 132.6 μmol/L, the absolute change criterion is theoretically more attainable than the relative change criterion. Therefore, the current study further examined the impact of these two criteria on test performance in patients with and without preexisting CKD (CKD subgroup vs. non-CKD subgroup), which is approximately equivalent to those with a baseline SCr level greater than or less than 132.6 μmol/L. When the absolute change criterion was applied, the renal recovery rate was 85.6% (394/446) in the non-CKD subgroup, which was higher than the 81.5% (243/298) in the CKD subgroup (*p* = 0.010). When the relative change criterion was utilized, the non-CKD subgroup demonstrated a significantly lower recovery rate of 76.9% (343/446) compared with the 85.6% recovery rate when the absolute change criterion was used (*p* < 0.001). In contrast, the CKD subgroup exhibited a recovery rate of 82.9% (247/298), similar to that obtained with the absolute change criterion. These findings support the hypothesis that the absolute change criterion is more attainable than the relative change criterion in patients without preexisting CKD. Consequently, in the entire cohort of 744 patients, the renal recovery rate was higher when the absolute change criterion was used than when the relative change criterion was used (85.6 vs. 79.3%, *p* = 0.001). As anticipated, the PPVs of the three tests for the entire patient cohort were greater when the absolute change criterion was used than when the relative change criterion was used.

The rationale for adopting two cutoff values for the sUCr/Osm test is as follows: (1) Both values demonstrated comparable Youden’s index results. In predicting renal recovery three months posthospitalization for acute kidney injury (AKI), defined as a SCr level increase of less than 26.5 μmol/L, the sUCr/Osm test with a cutoff value of 8.84 had a sensitivity of 0.881 and a specificity of 0.206, whereas the test with a cutoff of 21.22 had a sensitivity of 0.264 and a specificity of 0.785 (Table 3). (2) A tradeoff between sensitivity and specificity was observed when comparing test performance at these cutoff values, warranting further investigation. Using the Youden index method, the sUCr/Osm8.84 test demonstrated enhanced sensitivity for identifying patients likely to recover, whereas the sUCr/Osm21.22 test emphasized specificity and higher positive predictive values for discriminating recovery. Notably, the left reference limit for sUCr/Osm, typically observed in 95% of the general adult population, is 7.07 [10]. A sUCr/Osm value exceeding 8.84 in AKI patients indicates potential recovery in the urinary creatinine excretion rate. Moreover, when the mean sUCr/Osm value in a general adult population is 16.8 [10], a higher-than-average value, such as one exceeding 21.22, suggests an increased likelihood of SCr reversal through accelerated urinary creatinine excretion. These findings support the hypothesis that elevated sUCr/Osm values may serve as indicators of increased urinary creatinine excretion and an increased probability of renal recovery [11].

The associations of preexisting comorbidities, such as CKD, DM, and CHF, with nonrecovery following AKI have been thoroughly reviewed elsewhere [32] and reinforced in the current study. Further investigations were conducted to explore the potential additive effects of combining urinary biomarkers and underlying coexisting diseases in the prediction of renal recovery. The results revealed only marginal improvement (data not shown). While surgical admission has been found to be associated with higher renal recovery rates [16], no significant association was found between the specified insults and renal recovery rates in the current study. Given the multifactorial contributions and lack of detailed exposure information for each specified insult or event (e.g., contrast use, cardiac event, sepsis, major surgery, ICU stay, and ACE/ARB/NSAID use), it is reasonable to posit that the gross categorization into each clinical phenotype in the current study does not ensure that only one pathophysiological pathway is responsible, resulting in a particular pattern of outcomes for conclusive comparison.

The relationship between renal recovery and the use of loop diuretics remains uncertain. Loop diuretics may directly contribute to the development of acute interstitial nephritis [33]; however, an alternative hypothesis suggests that their use reflects the presence and severity of underlying cardiac conditions. Further analysis revealed that loop diuretic use was greater in patients with preexisting cardiac conditions (CHF or coronary artery disease)than in those without such conditions, both in the entire cohort (50.2% vs. 19.8%, *p* < 0.001) and in the CKD subgroup (65.7% vs. 34.0%, *p* < 0.001). When the absolute change criterion was applied, a greater proportion of patients with cardiac conditions and loop diuretic use achieved nonrecovery compared with the other patients in the entire cohort (23.4% vs. 10.8%, *p* < 0.001) or in the CKD subgroup (23.5% vs. 14.5%, *p* = 0.046). Similarly, with respect to the relative change criterion, nonrecovery was greater in patients with cardiac conditions and loop diuretic use in the entire patient cohort (29.4% vs. 17.2%, *p* < 0.001) or in the CKD subgroup (23.5% vs. 12.0%, *p* = 0.009) than in the other groups. These findings suggest that an interaction between loop diuretic use and underlying cardiac conditions is associated with reduced renal recovery. Further studies are needed to ascertain the causal relationships among underlying cardiac conditions, loop diuretic use, and renal recovery.

A key feature of sUCr/Osm is its independence from urine volume or concentration, rendering urine osmolality per se irrelevant to its interpretation. Acute loop diuretic use increases sodium excretion, lowering sUCr/Osm values, whereas chronic use has minimal impact due to the new establishment of sodium/electrolyte balance and volume homeostasis [34]. This study analyzed medication data, including loop diuretics, from prescriptions within 30 days before the index outpatient visit, which was approximately three months post-AKI diagnosis. Medications used prior to AKI onset or during spot urine sample collection were excluded because of limited periepisodic data. Given the study’s focus on three-month renal recovery, recent medication use within the last month was prioritized, and a separate analysis excluding chronic loop diuretic users was deemed unnecessary owing to the stability achieved in chronic administration.

One limitation of the current study is the exclusion of severe cases that required dialysis or resulted in mortality within three months following in-hospital AKI. Additionally, the lack of urine output data precludes the exclusion of cases with sufficient sUCr/Osm but inadequate urine output for creatinine and osmole excretion. The absence of comprehensive in-hospital management data also precludes the examination of their impact on renal recovery. As the urinary biomarkers and UCr/Osm were obtained within four days post-AKI diagnosis in the ASSESS-AKI study, their applicability for predicting early renal recovery within seven days was not analyzed. Moreover, given the renoprotective benefits of SGLT2is and their widespread use, further research is necessary to ascertain the impact of SGLT2i use on sUCr/Osm tests. SGLT2i-induced urinary glucose excretion adds 75–375 mmol of osmoles in urine per day [35], potentially lowering the left-sided reference limit for sUCr/Osm and the cutoffs for predicting renal recovery in SGLT2i users compared with nonusers. However, in cases where the eGFR falls below 15 mL/min/1.73 m^2^, the extent of induced glucosuria on the osmolar load remains to be elucidated [36]. Notably, this exploratory study evaluated dual cutoff points – rather than a single threshold – to balance sensitivity and specificity [37], thereby alleviating unnecessary anxiety about AKI recovery and reducing extensive testing.

In conclusion, urinary biomarkers such as UMOD may be applicable for both prediagnosis of AKI and predicting renal recovery, regardless of absolute or relative SCr level changes and preexisting CKD. Given their comparable test performance and greater accessibility, sUCr/Osm tests may be more practical for routine use. The sUCr/Osm8.84 test is sensitive for screening, whereas the sUCr/Osm21.22 test is specific for further discriminating potential renal recovery. Further prospective studies are needed to elucidate the precise clinical applications of these biomarkers.

## Data Availability

The dataset utilized in this secondary analysis study was consolidated from the primary datasets accessible upon request from the National Institute of Diabetes and Digestive and Kidney Diseases (NIDDK) Central Repository (https://repository.niddk.nih.gov/home/).
